# Deepoxy-deoxynivalenol (DOM-1), a derivate of deoxynivalenol (DON), exhibits less toxicity on intestinal barrier function, *Campylobacter jejuni* colonization and translocation in broiler chickens

**DOI:** 10.1186/s13099-021-00440-6

**Published:** 2021-07-03

**Authors:** Daniel Ruhnau, Claudia Hess, Barbara Doupovec, Bertrand Grenier, Dian Schatzmayr, Michael Hess, Wageha Awad

**Affiliations:** 1 Clinic for Poultry and Fish Medicine, Department for Farm Animals and Veterinary Public Health, University of Veterinary Medicine, Veterinärplatz 1, 1210 Vienna, Austria; 2 BIOMIN Research Center, Technopark 1, Tulln, Austria

**Keywords:** Deoxynivalenol (DON), Deepoxy-deoxynivalenol (DOM-1), *Campylobacter jejuni*, Colonization, Translocation, Intestinal permeability, Ussing chamber, Broiler chickens

## Abstract

**Background:**

Intestinal epithelial cells are challenged by mycotoxins and many bacterial pathogens. It was previously shown that the mycotoxin deoxynivalenol (DON) as well as *Campylobacter (C.) jejuni* have a negative impact on gut integrity. Recently, it was demonstrated that DON increased the load of *C. jejuni* in the gut and inner organs. Based on this finding, it was hypothesized the DON metabolite (deepoxy-deoxynivalenol, DOM-1) should be able to reduce the negative effects of DON on colonization and translocation of *C. jejuni* in broilers, since it lacks the epoxide ring, which is responsible for the toxicity of DON.

**Methods:**

A total of 180 broiler chickens were housed in floor pens on wood shavings with feed and water provided ad libitum. Birds were divided into six groups (n = 30 with 5 replicates/group): 1. Control, 2. DOM-1, 3. DON, 4. DOM-1 + *C. jejuni*, 5. DON + *C. jejuni*, 6. *C. jejuni*. At day 14, birds of groups 4, 5 and 6 were orally inoculated via feeding tube (gavage) with 1-ml of a PBS suspension containing 1 × 10^8^ CFU of *C. jejuni* NCTC 12744. The performance parameters: body weight (BW), body weight gain (BWG), and feed intake of the birds were determined. At 7, 14, and 21 days post infection, samples from liver, spleen, duodenum, jejunum and cecum were aseptically collected and processed for bacteriological investigations*.* Finally, at each killing time point, segments of duodenum, jejunum and cecum were harvested and prepared for Ussing chamber studies to measure the paracellular mannitol fluxes.

**Results:**

A significant decrease in body weight was observed for chickens receiving the DON diet with or without *C. jejuni* compared to the other groups. Furthermore, it was found that the co-exposure of birds to DON and *C. jejuni* resulted in a higher *C. jejuni* load not only in the gut but also in liver and spleen due to increased paracellular permeability of the duodenum, jejunum and cecum. On the contrary, DOM-1 supplementation in the feed improved the birds’ performance and led to a better feed conversion ratio throughout the trial. Furthermore, DOM-1 did not negatively affect gut permeability and decreased the *C. jejuni* counts in the intestine and internal organs.

**Conclusion:**

Altogether, the presence of DOM-1 in the feed as a result of the enzymatic biotransformation of DON leads to a lower *C. jejuni* count in the intestine and better feed conversion ratio. Moreover, this study demonstrates that the detoxification product of DON, DOM-1, does not have negative effects on the gastrointestinal tract and reduces the *Campylobacter* burden in chickens and also the risk for human infection.

## Background

Despite years of research, and the integration of appropriate agricultural and manufacturing practices in the food chain, mycotoxin burden remains a global problem [1]. Substantial economic losses are associated with mycotoxin contamination of feed due to impaired animal health, welfare and productivity [2]. Deoxynivalenol (DON) is a trichothecene and one of the most prevalent mycotoxins in the world. Chemically, trichothecenes belong to the group of sesquiterpenoids containing the 12, 13 epoxide group, considered to be critical for their toxicity. Due to the damaging effect of DON on the gut epithelium it can be expected that the toxin decreases the resistance of the gut to infectious agents. Indeed, it was found that the presence of mycotoxins during an infection potentiated the clinical signs of disease in cattle [3], pigs [4] and chickens [5]. Furthermore, it was shown in vitro that DON increased the translocation of a pathogenic *Escherichia coli* strain via the intestinal epithelial cell monolayer (IPEC-1) [6]. Recently, it was also demonstrated that feeding of DON is a predisposing factor for the development of necrotic enteritis in broiler chickens due to the negative influence of the mycotoxin on the epithelial barrier, which causes an increased intestinal nutrient availability for clostridial proliferation [5]. In agreement with this we found that the presence of DON in the broiler diet during an infection with *Campylobacter* (C.) *jejuni* lead to an increase in intestinal permeability and translocation of bacteria to inner organs with a decrease in birds’performance [7].

*C. jejuni* is the most prevalent food-borne pathogen, primarily associated with poultry. The relationship between *C. jejuni* and the chicken was originally thought to be commensal. Based upon recent findings, however, it was demonstrated that *C. jejuni* exacerbates the intestinal paracellular permeability with consequences on the translocation of bacteria to inner organs as well as a negative influence on the bird performance following experimental infections [8–13]. The high prevalence together with antibiotic-resistant *Campylobacter* strains [14, 15] underlines the importance to find intervention strategies to decrease *Campylobacter* burden in chickens and to minimize campylobacteriosis in humans. Many studies have been published on the use of competitive exclusion to control *Campylobacter* with contradictory outcome and until today there is no commercial product available that shows good results [16–19].

In case of DON, it was demonstrated in various trials that a microbial feed additive with de-epoxidation activity was able to negate the toxic effects induced by this mycotoxin in chickens [20–24]. Mycotoxin deactivators are capable to transform DON to non-toxic metabolites such as deepoxy-deoxynivalenol (DOM-1), 3-keto-DON, 3-epi-DON due to enzymatic biotransformation [25]. Widely used deactivators favouring the prevalence of DOM-1 support the idea to investigate consequences on gut health and *C. jejuni* colonization. The aforementioned metabolite has not yet been investigated in combination with a pathogen such as *C. jejuni* and remain to be elucidated. We therefore hypothesized that the dietary inclusion of the DON metabolite DOM-1 instead of DON could alleviate DON-related effects on performance, gut permeability and *C. jejuni* colonization. Therefore, the objective of the present study was to investigate the effect of the dietary addition of the DON metabolite (DOM-1) to broiler feed on the colonization of *C. jejuni* in the gut as well as the translocation of *C. jejuni* to inner organs in comparison to DON. Furthermore, the epithelial paracellular permeability in different intestinal parts which is possibly affected by the presence of DON/DOM-1 in combination with *C. jejuni*, was determined by applying the Ussing chamber technique.

## Results

### Comparative effect of DOM-1 and DON on zootechnical performance

**Fig. 1:**
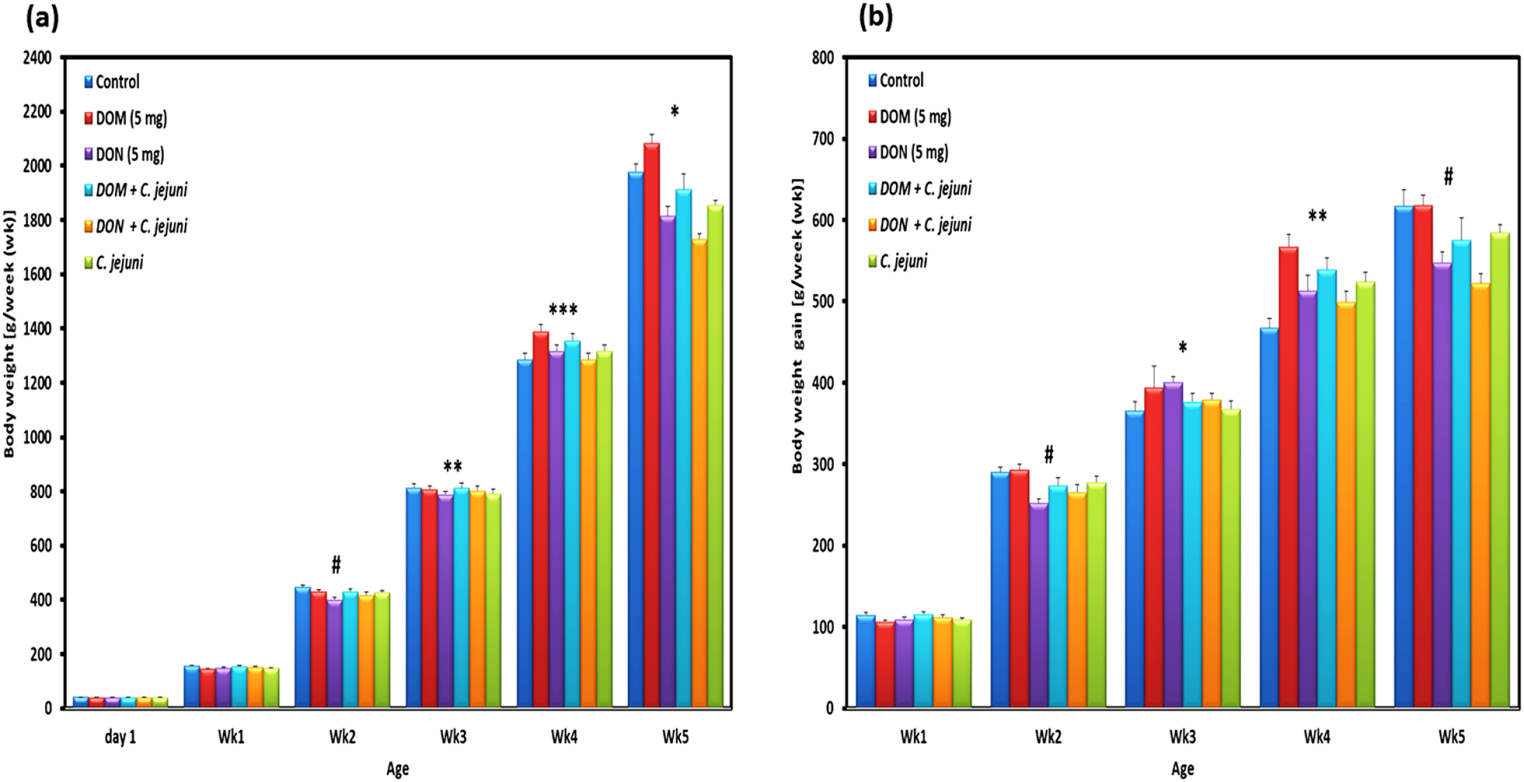
Exposure of broilers to deoxynivalenol and deepoxy-deoxynivalenol with/without *C. jejuni* and consequences on **a** body weight (BW) and **b** body weight gain (BWG). Results are presented as mean and standard error of mean (SEM). Asterisks mark differences with *P* ≤ 0.1 (^#^), *P* ≤ 0.05 (*), or *P* ≤ 0.001 (***)

**Fig. 2:**
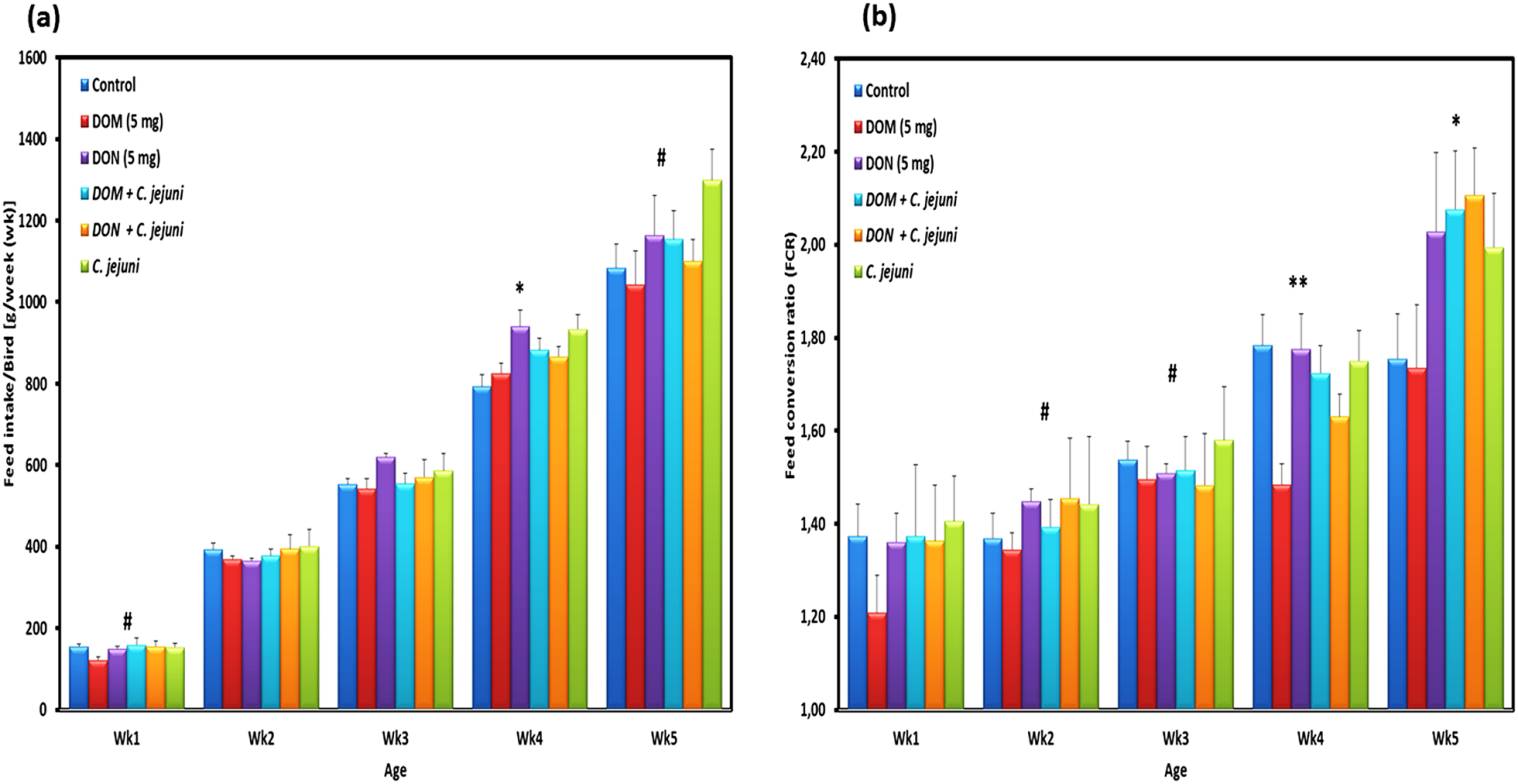
Comparative effect of the co-exposure to deoxynivalenol and deepoxy-deoxynivalenol with *C. jejuni* on **a** feed intake (FI) and **b** feed conversion rate (FCR). Results are presented as mean and SEM. Asterisks mark differences with *P* ≤ 0.1 (^#^), or *P* ≤ 0.05 (*)

Performance parameters are depicted in Figs. 1 and 2. The initial body weight of broiler chicks did not differ (*P* > 0.05) among the groups at day 1. During the experiment, the mean body weight per bird was significantly different between groups (*P* < 0.05), except at week 2 (wk2) (Fig. 1a). The growth performance of birds in groups 3 and 5 was numerically decreased (*P* < 0.1) at wk2. At wk4 and wk5 birds in group 2 had a significantly higher body weight (*P* < 0.01) compared with groups 1, 3 and 5, while birds in group 3 had a significantly (*P* < 0.001) lower body weight. Similarly, birds in groups 5 and 6 infected with *C. jejuni* had a significantly lower body weight (*P* < 0.05) compared with birds in the non-infected groups 1 and 2. Furthermore, comparing the average BW of the feeding groups at each week, the DOM-1 group had increased BW at wk2 and wk5 compared to that of the DON group (*P* = 0.040 and *P* = 0.008, respectively). Numerical changes in body weight gain (BWG) were demonstrated at wk2, wk5, and this effect reached the statistical significance (*P* < 0.05) at wk3 and wk4. Moreover, birds in group 2 had a numerically higher weight gain at wk2 and wk5 and a significant higher weight gain at wk4 in comparison to other groups (Fig. 1b). Additionally, the BWG was significantly increased for birds supplemented with DOM-1 at wk2, wk4 and wk5 compared to that of the DON group (*P* = 0.001, *P* = 0.051, and *P* = 0.005, respectively). The overall body weight gain was significantly (P = 0.007) lower (1750 ± 36 g) in group 3 compared with group 1 (1937 ± 27 g), while birds in group 2 had the highest weight gain (2043 ± 31 g). Furthermore, DOM-1 supplemented birds had a greater overall body weight gain than DON supplemented birds (*P* = 0.005).

The feed intake was not significantly different between groups during the first three weeks (Fig. 2a) and only slight differences were observed in the average daily feed intake between group 1 (85 g/bird/day) and group 2 (83 g/bird/day). An increase in the average feed intake from week 3 onwards was recorded in groups 3, 4, 5, and 6 compared to groups 1 and 2. This finding was significant at wk4 (P < 0.05) and remained at a numerical difference in wk5. Furthermore, a decreased feed intake was observed at wk4 for the DOM-1 feed group compared to that in the DON group (P = 0.018). In this context, feed conversion ratio (FCR) at wk4 and wk5 of infected birds in groups 4, 5, and 6 was higher compared with non-infected birds in groups 1, 2 and 3 (Fig. 2b). In addition, DOM-1 supplemented birds had a lower FCR than DON supplemented birds at wk4 and wk5 (*P* = 0.000, *P* = 0.033, respectively). Throughout the whole trial, the average feed conversion ratio was lower in group 2 compared with the other groups and this effect was significant at wk4 and wk5 (Fig. 2b), indicating that DOM-1 was able to improve the feed efficiency under the present experimental conditions.

### Comparative effect of DOM-1 and DON on colonization and translocation of *C. jejuni*

**Fig. 3:**
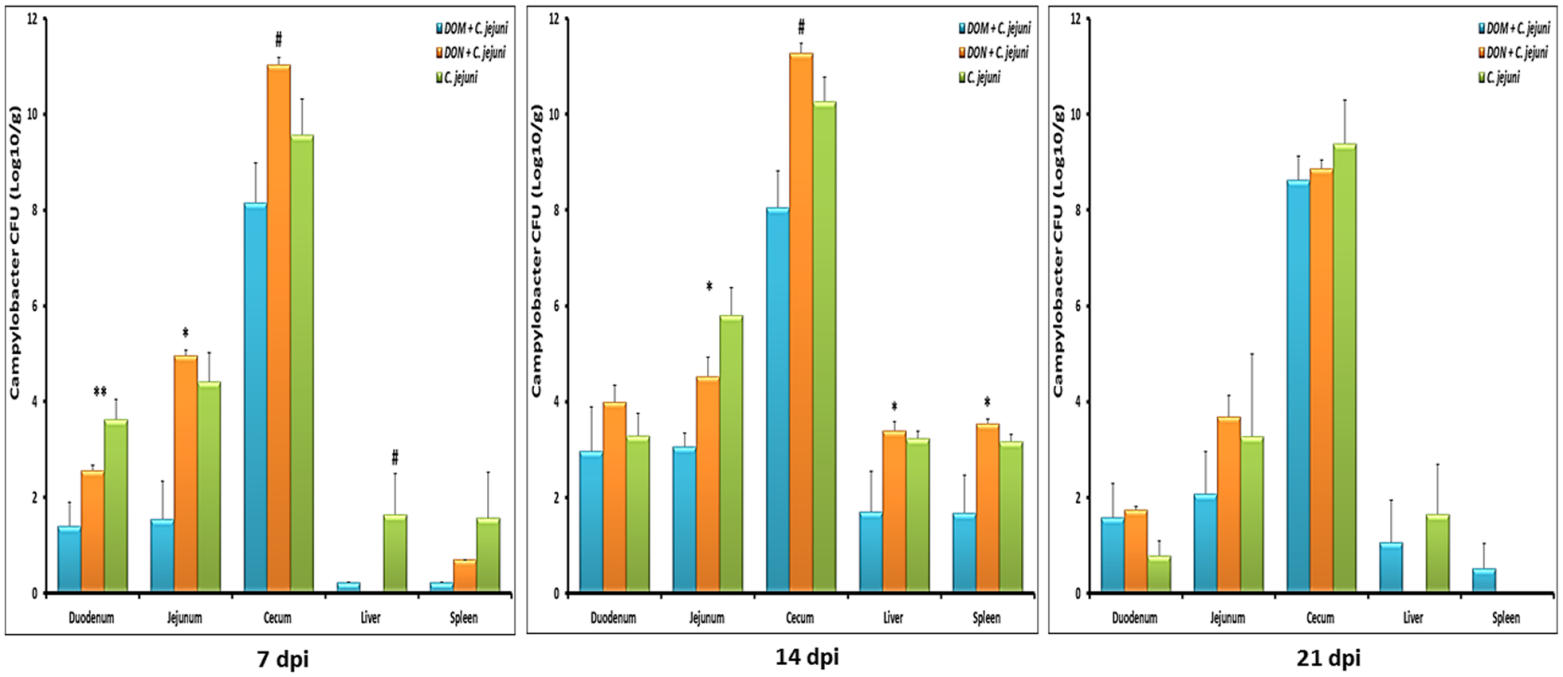
Comparative effect of the co-exposure to deoxynivalenol and deepoxy-deoxynivalenol with *C. jejuni* on the colonization and translocation of *Campylobacter* at different times post infection. Results are presented as mean and SEM (n = 5). Asterisks mark differences with *P* ≤ 0.1 (^#^), or *P* ≤ 0.05 (*)

Prior to the experimental infection, all birds were confirmed as *Campylobacter* free by cloacal swab samples taken from day-old birds and at 14 days of age. The *C. jejuni* load in the intestine and inner organs is presented in Fig. 3. In group 5, birds had higher loads of *C. jejuni* in the jejunum and cecum at 7 dpi compared to birds in group 6, however, at that time, no significant differences were found regarding the *C. jejuni* counts in liver and spleen of the birds fed different diets. At 14 dpi a significant increase of *C. jejuni* was found in caeca as well as in the liver and spleen of DON-fed birds (group 5). Furthermore, co-exposure of broiler chickens to DON and *C. jejuni* supported *C. jejuni* colonization in jejunum and cecum and induced translocation to the liver and spleen at 14 dpi and 21 dpi, indicating that the noticeable effect of the co-exposure is more prominent at later stages. The dietary inclusion of DOM-1 in group 4 decreased the load of *C. jejuni* in duodenum, jejunum and cecum significantly at 7 and 14 dpi. Moreover, a decreased load of *C. jejuni* in jejunum and cecum was observed at 7 dpi for the DOM-1 feed group compared to that in the DON group (P = 0.012 and *P* = 0.033, respectively). In line with that, at 14 dpi, DOM-1 decreased the translocation of *C. jejuni* to liver and spleen compared to that of the DON group (*P* = 0.037 and *P* = 0.016, respectively). Later on, no significant differences between groups were noticed although a certain tendency remained with lower *C. jejuni* counts in DOM-1 fed birds.

### Comparative effect of DOM-1 and DON on intestinal paracellular permeability

**Fig. 4:**
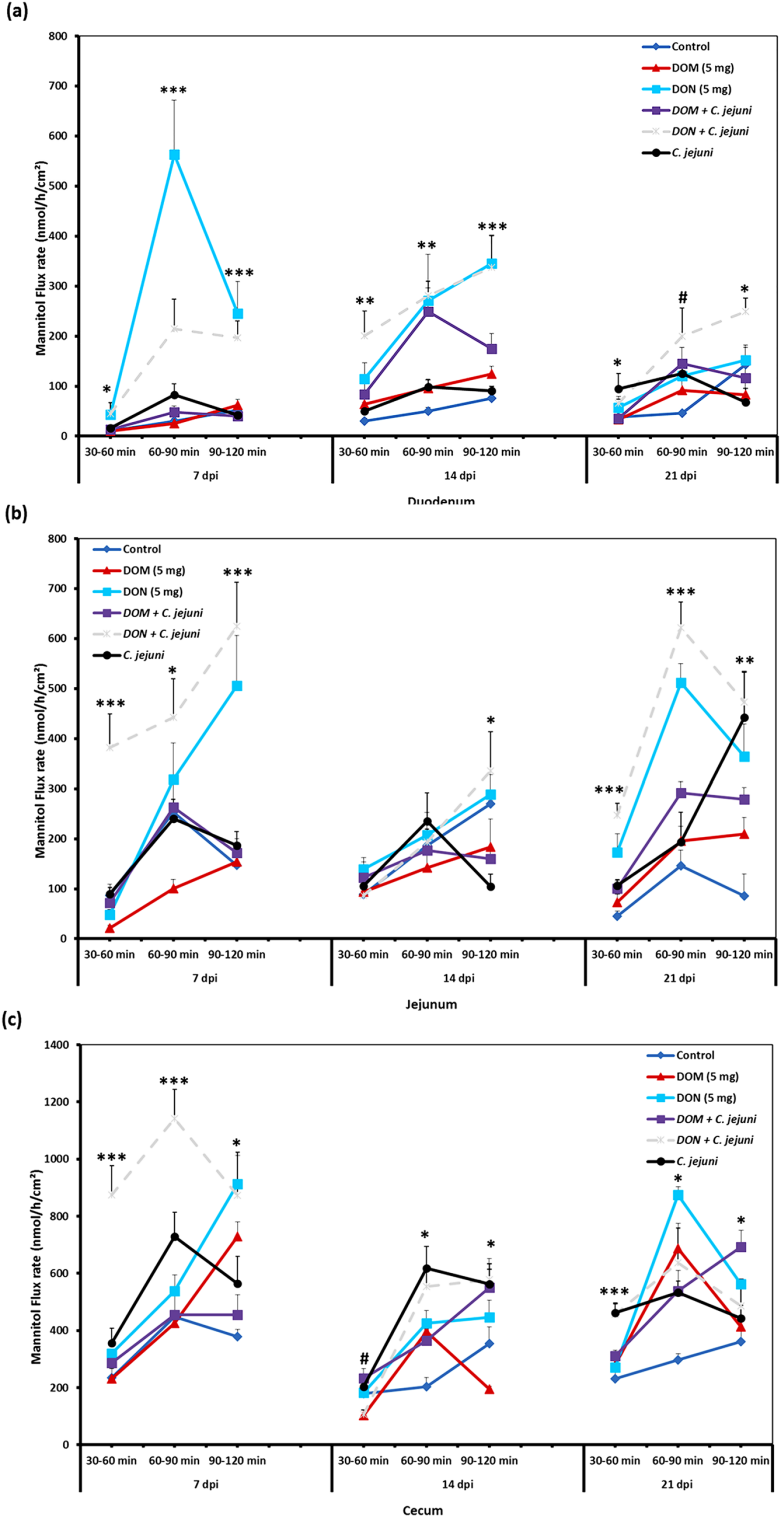
Comparative effect of the co-exposure to deoxynivalenol and deepoxy-deoxynivalenol with *C. jejuni* on the paracellular permeability in the duodenum (**a**), jejunum (**b**) and cecum (**c**) at different time points post infection. Mucosal to serosal flux (J_ms_) of the permeability marker ^14^C-mannitol were performed in Ussing chambers. Data are presented as the mean values and SEM (n = 5). Asterisks mark differences with P ≤ 0.1 (#), P ≤ 0.05 (*), P ≤ 0.01 (**), or P ≤ 0.001 (***)

The unidirectional mucosa-to-serosa permeability of ^14^C mannitol in duodenum, jejunum and cecum is shown in Fig. 4a–c. The findings revealed that DON caused a disruption of the intestinal epithelial barrier function and increased paracellular permeability. Similarly, the results showed that *C. jejuni* exposure induces a significant increase in the flux of ^14^C mannitol in all parts of the intestine. Consequently, this effect was particularly pronounced in birds co-exposed to DON and *C. jejuni* (group 5). During the baseline period (30–60 min), there were significant differences in the flux of the marker molecule in the duodenum, jejunum and cecum among the different groups at 7 dpi. Furthermore, during the second flux period (from 60 to 90 min), a continuous increase in ^14^C mannitol flux was found, most probably because of passive diffusion. It was obvious that the effect of DON was more persistent in the jejunum and cecum at 21 dpi. Furthermore, the co-exposure to DON and *C. jejuni* potentiates this negative effect on the permeability of the duodenum in all flux periods, which is indicated by a higher mannitol flux at 7 dpi (*P* < 0.05), 14 dpi (*P* < 0.001) and 21 dpi (*P* < 0.05) compared to the controls. Similarly, the results revealed that exposure with DON induced an increase in the flux of mannitol in the jejunum and cecum at 7 dpi, 14 dpi and 21 dpi. On the contrary, feeding of DOM-1 induced no significant changes in intestinal permeability and led to a comparable permeability of the chicken gut as in the control group. In addition, at 7 dpi, a decreased mannitol flux in the duodenum (P = 0.019, *P* = 0.000, and *P* = 0.000 during the first (30–60 min), second (60–90 min) and third (90–120 min) flux periods, respectively), and in the jejunum (*P* = 0.025 and *P* = 0.000, during the second and third flux periods, respectively) were observed for the DOM-1 fed group compared to that in the DON fed group. This difference in the mannitol flux between the DOM-1 fed group and DON fed group was more pronounced in the duodenum and jejunum at 7 dpi and 14 dpi (*P* < 0.01), but this difference did not reach statistical significance at 21 dpi which could be attributed to that DON is rapidly and efficiently absorbed in the upper gastrointestinal tract. Data showed that the impact of the dietary treatments on intestinal permeability varies highly between different gut sites, which likely reflects different mechanisms for the alterations of intestinal permeability of each intestinal segment.

## Discussion

Mycotoxin contamination of poultry feeds is a worldwide problem, as this can increase the incidence of disease and reduce production efficiency [26]. In general, the contamination of livestock feed with mycotoxins has a profound effect on animal welfare and productivity [27, 28]. Furthermore, contrary to pathogens exposure, there are no clear clinical signs of mycotoxin intoxication, as mycotoxins are normally present at low levels. However, they are able to damage epithelial tissue, increase intestinal permeability, and therefore may result in a weakened immune system or even death [29, 30, 31, 26].

Mechanisms how mycotoxins influence prokaryotes also began to emerge as an important area of future research perspectives [32, 33]. Recently, presented evidence indicates that DON can negatively affect the gut microbiota of either humans or animals [34, 35]. This, in turn, has led to a greater interest in understanding bacterial responses towards DON. Applying an experimental model for necrotic enteritis Antonissen et al. [5] found that broiler chickens fed a diet contaminated with 5 mg DON/kg feed were more prone to develop necrotic enteritis compared to chickens fed with the control diet. Previously, it was also shown that the co-exposure of broiler chickens to DON and *C. jejuni* supported the *C. jejuni* colonization in the gut at certain time points post infection, revealing that DON might provide a favorable condition for *Campylobacter* growth [7].

The high prevalence of *Campylobacter jejuni* in broilers combined with the fear to spread multi-drug resistance genes underlines its high importance from a socio-economic perspective. In consequence, many studies attempted to combat the burden of this bacterium in poultry in order to decrease the risk of human infection [36–39]. It has been shown that the use of competitive exclusion to control *Campylobacter* results in a contradictory outcome and until today there is no commercial product available with good efficacy [16, 18, 19]. Similarly, for DON, it was reported that mycotoxin deactivators were capable to transform DON into the non-toxic metabolite DOM-1 due to enzymatic biotransformation, thus decreasing the DON burden in the chicken. However, direct effects of purified DOM-1 on gut barrier and performance have never been assessed in chickens. Furthermore, no data are available about the interaction of DON or the non-toxic metabolite DOM-1 in context of an *C. jejuni* infection in broiler chickens, altogether the subject of the actual study*.*

Overall, the actual study demonstrated that the dietary inclusion of a non-toxic metabolite of DON, DOM-1, does not lead to negative effects in broiler chickens. Concurrently, birds fed with DOM-1 had a better performance compared to all other groups, as shown by the higher body weight and body weight gain, as well as an improved feed conversion ratio. The better performance in the DOM-1 -supplemented birds could be explained by that the non-toxic metabolite of DON claims several modes of action such as activation and supporting the liver function as well as enhancement of the immune system, metabolic enzyme activity and feed digestibility [40]. On the contrary, the BW and feed intake were negatively affected by DON at 5 mg/kg. The impaired growth performance results may be related to changes in gut physiology caused by the mycotoxin. Negative effects on nutrient digestibility and BW could be explained by suppressed villus length and a decrease in the nutrient absorption surface area in the jejunum [41]. Similarly, a linear decrease in feed intake and weight gain with increasing dietary proportions of DON-contaminated wheat in the diet of male broilers reared for 5 weeks was reported [42]. Currently, the guidance level for DON in the European Union in complete feed for poultry is set at 5 mg DON/kg feed (EC 2006). However, the toxic effects of DON in poultry depend not only on the dose but also on the length of exposure to DON as well as other factors, whereby the interactions with other dietary components that are affecting intestinal health may play a role [31]. Moreover, the inconsistency of the performance does not only rely on DON as such, but also on the growth potential and feed efficiency traits of birds. Consequently, it is important to re-evaluate threshold levels for DON in chicken feed which are reflected in the currently applicable guidance values (EC 2006).

In this context, the results of the actual study also indicated that DON increased the intestinal paracellular permeability as reported in previous studies [29, 7]. Similarly, effects of *C. jejuni* on the gut physiology of chickens have also been reported [12]. It was shown that these bacteria have a negative impact on the nutrient absorption indicating that a lower slaughter weight might probably be due to the reduction in the feed efficiency [13]. Furthermore, the occurrence of a leaky gut syndrome caused by *C. jejuni* is known to enhance bacterial translocation from the gut to internal organs [9, 11]. The study also revealed that the co-exposure to DON and *C. jejuni* potentiates a significant increase in paracellular permeability. Interestingly, the intestinal permeability was not negatively influenced by the feeding of DOM-1 and led to comparable intestinal permeability as in the control group.

Effects of purified DOM-1 on the intestine have never been tested. However, nutritional strategies including bacteria/enzyme transforming DON to deepoxy-DON reduced the occurrence and extent of intestinal lesions resulting in the same zootechnical performance as the control animals [21, 43, 44]. Recently, an increase in the colonization and translocation of *C. jejuni* and *E. coli* could be demonstrated in birds fed with DON, again confirmed in the actual study [7]. By contrast, feeding of DOM-1 reduced the colonization of all measured parts of the intestine with *C. jejuni* in a range of 1.5–3.0 log during the first two weeks post infection compared to the DON + *C. jejuni* treatment. Furthermore, it can also be hypothesized that DOM-1 may create a different intestinal milieu to *C. jejuni* at a certain time point postinfection. However, these findings warrant additional studies to explain how DON and DOM-1 could affect (directly or indirectly) the level of a prokaryote such as *Campylobacter* in chickens. The reduced colonization of *C. jejuni* due to DOM-1 supplementation could be explained by the fact that DOM-1 could have beneficial effects on the microbial populations and metabolic end products (short-chain fatty acids, SCFAs) in the intestine, since it was thought that a more stable microbiota might prevent the colonization of pathogens [45]. Finally, the results of this study can be useful in understanding how gram-negative bacteria respond to DON and DOM-1, however the bacterial response at the transcriptome level needs further investigation.

## Conclusion

In summary, results of the current study showed that the presence of DOM-1 in the feed as a result of the enzymatic biotransformation of DON does not have toxic effects on zootechnical parameters and intestinal permeability. The results demonstrated that the dietary inclusion of DOM-1 in broiler feed together with an *C. jejuni* infection decreased bacterial load in the gut and reduced *Campylobacter* dissemination to inner organs which might be due to DOM-1 -related changes in host physiology and intestinal permeability in comparison to DON. Besides, the dietary supplementation of DOM-1 to the infected birds effectively alleviated the intestinal alterations caused by *C. jejuni* and compensated negative effects on permeability. Moreover, an improvement in birds’ performance was observed by DOM-1 feeding. Feed technologies, reducing not only the detrimental effects of DON in chicken feed but also the *Campylobacter* burden, display a significant impact on animal welfare and public health.

## Material and methods

### Birds, treatment groups and bacteriological study

**Fig. 5:**
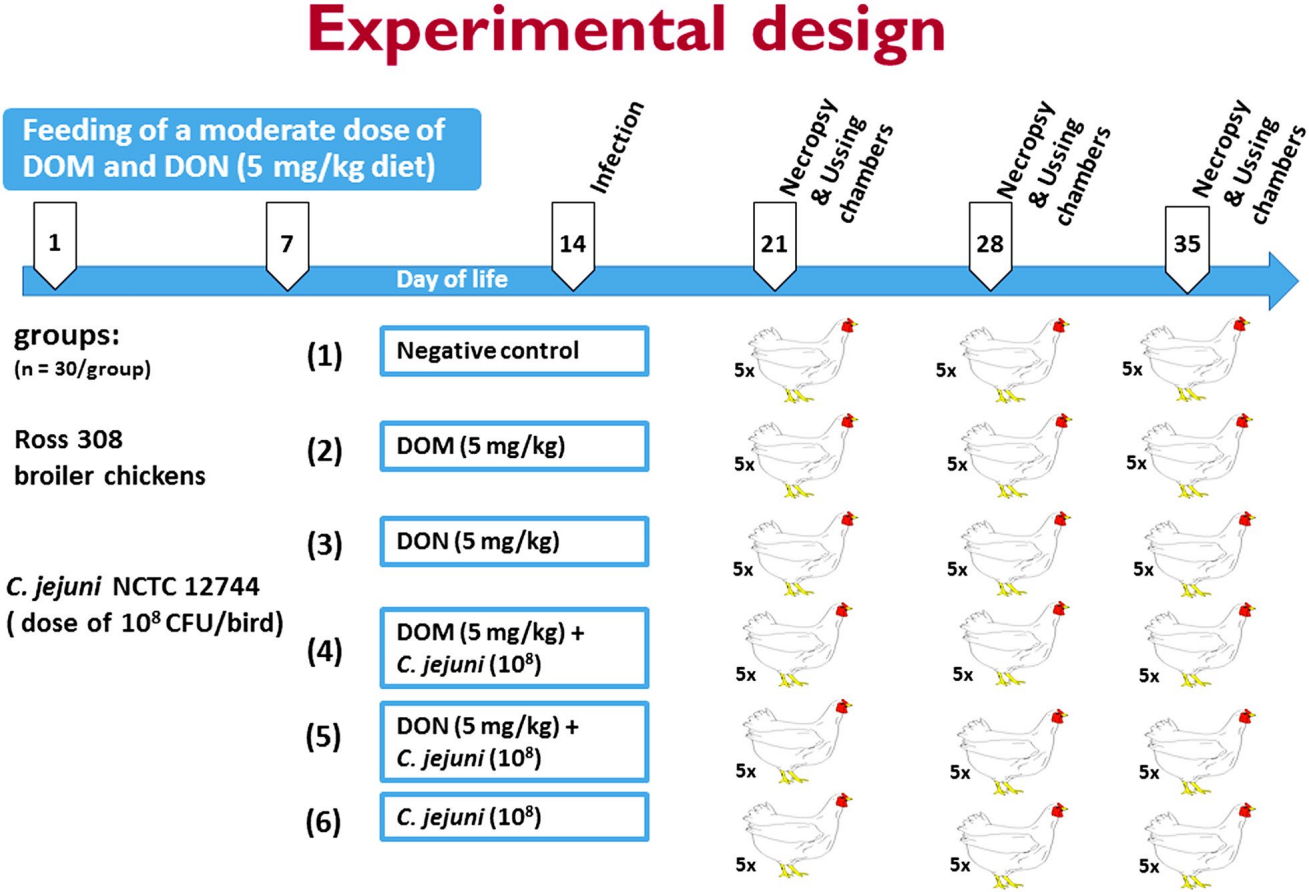
Schematic outline of the experimental design. In the control group, birds were left untreated and fed the basal diet with no supplementation. In the DON and DOM-1 treated groups, birds were fed for 5 weeks with either DON or DOM-1. On day 14 of age, each bird within groups 4, 5 and 6 was orally inoculated into the crop with 10^8^ CFU of *C. jejuni* (NCTC 12744)

A total of 180 one-day–old broiler chicks were obtained from a commercial hatchery (Ross-308; Geflügelhof Schulz, Lassnitzhöhe, Austria) and divided into six treatment groups, each with an identical set up (n = 30 birds with 5 replicates/ group): 1. negative control (basal diet), 2. DOM-1 supplemented diet (5 mg/kg feed), 3. DON contaminated diet (5 mg/kg feed), 4. DOM-1 supplemented diet (5 mg/kg feed) + *C. jejuni* infection, 5. DON contaminated diet (5 mg/kg feed) + *C. jejuni* infection, 6. positive control (*C. jejuni* infection + basal diet) (Fig. 5). On the day of hatch, chicks were tagged, weighed and put into floor pens equipped with fresh wooden shavings litter under strict conditions of biosecurity. Light was provided in a 16:8 light/dark cycle. Temperature was kept at 35 °C during the first days of life provided via red light spot lamps and later reduced to 25 °C with the age of birds. Humidity was at an interval between 55 and 60%. Feed and water were provided ad libitum. The birds were fed for 5 consecutive weeks with either contaminated diets with 5 mg DON/kg feed, or basal diet supplemented with 5 mg DOM-1/kg feed (BIOMIN Research Center, Technopark 1, 3430 Tulln, Austria), or only basal diets (control, non-contaminated diet during the starter and grower periods). The control diet was prepared with non-contaminated wheat. The mycotoxin contaminated diet was prepared by replacing “non-contaminated” control wheat with DON contaminated wheat. Moreover, the 5 mg DON/kg feed in this study is the currently applicable EU guidance value of DON contamination (5 mg DON/kg poultry feed) (EC, 2006). Consequently, the used feeding model is relevant to mimic the field situation. The composition of the diet comprised maize, wheat, soy, soybean meal, soybean oil, and rapeseed oil. Additionally, a premix of vitamins, minerals, mono calcium phosphate, and salt was supplemented. Feeds were provided by Biomin Holding GmbH (Tulln, Austria). Starter diet was fed for 9 days, followed by the grower diet from day 10 until 35 days of age. Representative feed samples for each group were analyzed for determining the concentration of DON, DOM-1 and other mycotoxins in the diets.

Birds were monitored daily for any adverse effects or clinical signs. Performance parameters monitored during the trial included body weight of each bird, which was taken at the first day of the trial and afterwards weekly, body weight gain was determined weekly and feed consumption was calculated weekly for each group and accordingly feed conversion ratio was measured. Cloacal swabs were taken on day one and prior to infection. These swabs were directly streaked onto modified charcoal-cefoperazone-deoxycholate agar (mCCDA Oxoid, Hampshire, UK), and incubated at 41.5 °C under microaerophilic conditions (Genbox microaer, BioMérieux, Vienna, Austria). On day 14 of age, chicks from groups 4, 5 and 6 were orally inoculated into the crop with 1 × 10^8^ cfu/ml *C. jejuni* NCTC 12744 using a syringe connected to a stainless-steel cannula.

At 7, 14- and 21-days post infection, five birds from each group were randomly selected and euthanized for sampling. Gross pathological examination was followed by collecting tissue samples from liver, spleen, duodenum, jejunum and cecum for bacterial enumeration. These samples were homogenized (Ultra-Turrax IKA, Staufen, Germany) in 1:10 (wt:vol) PBS (phosphate buffered saline, Life Technologies Limited, Paisley, UK), followed by a preparation of serial ten-fold dilution and direct plating in duplicate on CASA agar (BioMérieux, Vienna, Austria). CFU counts were determined by calculating the mean value of both plates.

### Intestinal paracellular permeability

At each killing time point, segments of duodenum, jejunum and cecum (2 replicates/segment/bird) were harvested from five birds for five birds per group, placed in ice-cooled buffer solution and prepared for Ussing chamber studies to measure the paracellular mannitol fluxes. Epithelial layers had an exposed tissue surface of 1.1 cm^2^ and were incubated with 12 ml of buffer solution on either side under short-circuit conditions. Tissue stabilization was followed by the addition of the radioactive tracer, ^14^C-mannitol (0.1 mCi/ml; Hartmann Analytic, Steinriedendamm 15 Braunschweig, Germany), to the mucosal solution. After a 30-min equilibration period, standards (0.1-mL) were collected from the mucosal side of each chamber and 30-min flux periods were established by taking 0.6-mL samples from the serosal compartment, four times in total. All procedures were performed as described by Awad et al. [29]. Finally, the presence of ^14^C-mannitol was detected by measuring β emission in a liquid scintillation counter after addition of up to 5 mL a liquid scintillation fluid to all samples (Ultima Gold, Perkin Elmer, MA, USA).

### Statistical analysis

Data are presented as means with standard error of mean (SEM). To evaluate the normality Kolmogorov–Smirnov´s test was utilized. A multivariate general linear model, ANOVA, Duncan´s multiple range test and LSD were performed to analyze performance, bacterial translocation and mannitol flux data. Data were analyzed by IBM SPSS Statistics 24 software for Windows (Chicago, IL, USA).

## Data Availability

The raw data supporting the conclusions of this article will be made available by the authors, without undue reservation.

## References

[1] Logrieco AF, Miller JD, Eskola M, Krska R, Ayalew A, Bandyopadhyay R, Battilani P, Bhatnagar D, Chulze S, De Saeger S, Li P, Perrone G, Poapolathep A, Rahayu ES, Shephard GS, Stepman F, Zhang H, Leslie JF (2018). The mycotox charter: increasing awareness of, and concerted action for minimizing mycotoxin exposure worldwide. Toxins.

[2] Pitt JI, Miller JD (2017). A concise history of mycotoxin research. J Agric Food Chem.

[3] Baines D, Sumarah M, Kuldau G, Juba J, Mazza A, Masson L (2013). Aflatoxin, fumonisin and Shiga toxin-producing Escherichia coli infections in calves and the effectiveness of celmanax®/dairyman’s choice^TM^ applications to eliminate morbidity and mortality losses. Toxins.

[4] Vandenbroucke V, Croubels S, Verbrugghe E, Boyen F, De Backer P, Ducatelle R, Rychlik I, Haesebrouck F, Pasmans F (2009). The mycotoxin deoxynivalenol promotes uptake of Salmonella Typhimurium in porcine macrophages, associated with ERK1/2 induced cytoskeleton reorganization. Vet Res.

[5] Antonissen G, Van Immerseel F, Pasmans F, Ducatelle R, Haesebrouck F, Timbermont L, Verlinden M, Janssens GP, Eeckhaut V, Eeckhout M, De Saeger S, Hessenberger S, Martel A, Croubels S (2014). The mycotoxin deoxynivalenol predisposes for the development of *Clostridium perfringens*-induced necrotic enteritis in broiler chickens. PLoS ONE..

[6] Pinton P, Nougayrède JP, Del Rio JC, Moreno C, Marin DE, Ferrier L, Bracarense AP, Kolf-Clauw M, Oswald IP (2009). The food contaminant deoxynivalenol, decreases intestinal barrier permeability and reduces claudin expression. Toxicol Appl Pharmacol.

[7] Ruhnau D, Hess C, Grenier B, Doupovec B, Schatzmayr D, Hess M, Awad WA (2020). The Mycotoxin Deoxynivalenol (DON) Promotes *Campylobacter jejuni* multiplication in the intestine of broiler chickens with consequences on bacterial translocation and gut integrity. Front Vet Sci..

[8] Awad WA, Aschenbach JR, Ghareeb K, Khayal B, Hess C, Hess M (2014). *Campylobacter jejuni* influences the expression of nutrient transporter genes in the intestine of chickens. Vet Microbiol..

[9] Awad WA, Dublecz F, Hess C, Dublecz K, Khayal B, Aschenbach JR, Hess M (2016). *Campylobacter jejuni* colonization promotes the translocation of Escherichia coli to extra-intestinal organs and disturbs the short-chain fatty acids profiles in the chicken gut. Poult Sci.

[10] Awad WA, Hess C, Hess M (2018). Re-thinking the chicken-Campylobacter jejuni interaction: a review. Avian Pathol.

[11] Awad WA, Mann E, Dzieciol M, Hess C, Schmitz-Esser S, Wagner M, Hess M (2016). Age-related differences in the luminal and mucosa-associated gut microbiome of broiler chickens and shifts associated with *Campylobacter jejuni* infection. Front Cell Infect Microbiol.

[12] Awad WA, Ruhnau D, Hess C, Hess M (2020). *Campylobacter jejuni* increases the paracellular permeability of broiler chickens in a dose-dependent manner. Poult Sci.

[13] Awad WA, Smorodchenko A, Hess C, Aschenbach JR, Molnár A, Dublecz K, Khayal B, Pohl EE, Hess M (2015). Increased intracellular calcium level and impaired nutrient absorption are important pathogenicity traits in the chicken intestinal epithelium during *Campylobacter jejuni* colonization. Appl Microbiol Biotechnol.

[14] Quinn T, Bolla JM, Pagès JM, Fanning S (2007). Antibiotic-resistant Campylobacter: could efflux pump inhibitors control infection?. J Antimicrob Chemother.

[15] Wang Y, Zhang M, Deng F, Shen Z, Wu C, Zhang J, Zhang Q, Shen J (2014). Emergence of multidrug-resistant *Campylobacter* species isolates with a horizontally acquired rRNA methylase. Antimicrob Agents Chemother.

[16] Ghareeb K, Awad WA, Mohnl M, Porta R, Biarnés M, Böhm J, Schatzmayr G (2012). Evaluating the efficacy of an avian-specific probiotic to reduce the colonization of *Campylobacter jejuni* in broiler chickens. Poult Sci.

[17] Robyn J, Rasschaert G, Pasmans F, Heyndrickx M (2015). Thermotolerant *Campylobacter* during broiler rearing: risk factors and intervention. Compr Rev Food Sci Food Saf.

[18] Santini C, Baffoni L, Gaggia F, Granata M, Gasbarri R, Di Gioia D, Biavati B (2010). Characterization of probiotic strains: an application as feed additives in poultry against *Campylobacter jejuni*. Int J Food Microbiol.

[19] Willis WL, Reid L (2008). Investigating the effects of dietary probiotic feeding regimens on broiler chicken production and Campylobacter jejuni presence. Poult Sci.

[20] Awad WA, Böhm J, Razzazi-Fazeli E, Ghareeb K, Zentek J (2006). Effect of addition of a probiotic microorganism to broiler diets contaminated with deoxynivalenol on performance and histological alterations of intestinal villi of broiler chickens. Poult Sci.

[21] Awad WA, Böhm J, Razzazi-Fazeli E, Hulan HW, Zentek J (2004). Effects of deoxynivalenol on general performance and electrophysiological properties of intestinal mucosa of broiler chickens. Poult Sci.

[22] Awad WA, Ghareeb K, Dadak A, Hess M, Böhm J (2014). Single and combined effects of deoxynivalenol mycotoxin and a microbial feed additive on lymphocyte DNA Damage and Oxidative Stress in Broiler Chickens. PLoS ONE.

[23] Ghareeb K, Awad WA, Soodoi C, Sasgary S, Strasser A, Böhm J (2013). Effects of feed contaminant deoxynivalenol on plasma cytokines and mRNA expression of immune genes in the intestine of broiler chickens. PLoS ONE..

[24] Ghareeb K, Awad WA, Sid-Ahmed OE, Böhm J (2014). Insights on the host stress, fear and growth responses to the deoxynivalenol feed contaminant in broiler chickens. PLoS One..

[25] Vanhoutte I, De Mets L, De Boevre M, Uka V, Di Mavungu JD, De Saeger S, De Gelder L, Audenaert K (2017). Microbial detoxification of Deoxynivalenol (DON), Assessed via a Lemna minor L. Bioassay, through Biotransformation to 3-epi-DON and 3-epi-DOM-1. Toxins..

[26] Murugesan GR, Ledoux DR, Naehrer K, Berthiller F, Applegate TJ, Grenier B, Phillips TD, Schatzmayr G (2015). Prevalence and effects of mycotoxins on poultry health and performance, and recent development in mycotoxin counteracting strategies. Poult Sci.

[27] Gallo A, Giuberti G, Frisvad JC, Bertuzzi T, Nielsen KF (2015). Review on mycotoxin issues in ruminants: occurrence in forages, effects of mycotoxin ingestion on health status and animal performance and practical strategies to counteract their negative effects. Toxins.

[28] Magnin M, Travel A, Bailly J, Guerre P (2016). Effects of mycotoxins on health and performance in poultry. Prod Anim -Paris- Inst Natl la Rech Agron.

[29] Awad WA, Ruhnau D, Hess C, Doupovec B, Schatzmayr D, Hess M (2019). Feeding of deoxynivalenol increases the intestinal paracellular permeability of broiler chickens. Arch Toxicol.

[30] Döll S, Schrickx JA, Dänicke S, Fink-Gremmels J (2009). Deoxynivalenol-induced cytotoxicity, cytokines and related genes in unstimulated or lipopolysaccharide stimulated primary porcine macrophages. Toxicol Lett.

[31] Ghareeb K, Awad WA, Böhm J, Zebeli Q (2015). Impacts of the feed contaminant deoxynivalenol on the intestine of monogastric animals: poultry and swine. J Appl Toxicol.

[32] Hassan YI, He JW, Lepp D, Zhou T (2019). Understanding the bacterial response to mycotoxins: the transcriptomic analysis of deoxynivalenol-induced changes in *Devosia mutans* 17-2-E-8. Front Pharmacol.

[33] Payros D, Dobrindt U, Martin P, Secher T, Bracarense AP, Boury M, Laffitte J, Pinton P, Oswald E, Oswald IP (2017). The food contaminant deoxynivalenol exacerbates the genotoxicity of gut microbiota. MBio..

[34] Lucke A, Böhm J, Zebeli Q, Metzler-Zebeli BU (2018). Dietary deoxynivalenol contamination and oral lipopolysaccharide challenge alters the cecal microbiota of broiler chickens. Front Microbiol.

[35] Robert H, Payros D, Pinton P, Théodorou V, Mercier-Bonin M, Oswald IP (2017). Impact of mycotoxins on the intestine: are mucus and microbiota new targets?. J Toxicol Environ Health B Crit Rev.

[36] Arsi K, Donoghue AM, Woo-Ming A, Blore PJ, Donoghue DJ (2015). The efficacy of selected probiotic and prebiotic combinations in reducing Campylobacter colonization in broiler chickens. J Appl Poult Res.

[37] Buckley AM, Wang J, Hudson DL, Grant AJ, Jones MA, Maskell DJ, Stevens MP (2010). Evaluation of live-attenuated Salmonella vaccines expressing Campylobacter antigens for control of *C. jejuni* in poultry. Vaccine..

[38] El-Shibiny A, Scott A, Timms A, Metawea Y, Connerton P, Connerton I (2009). Application of a group II Campylobacter bacteriophage to reduce strains of *Campylobacter jejuni* and *Campylobacter coli* colonizing broiler chickens. J Food Prot.

[39] Kumar A, Drozd M, Pina-Mimbela R, Xu X, Helmy YA, Antwi J, Fuchs JR, Nislow C, Templeton J, Blackall PJ, Rajashekara G (2016). Novel anti-campylobacter compounds identified using high throughput screening of a pre-selected enriched small molecules library. Front Microbiol.

[40] Kiyothong K, Rowlinson P, Wanapat M, Khampa S (2012). Effect of mycotoxin deactivator product supplementation on dairy cows. Anim Product Sci.

[41] Awad WA, Hess M, Twarużek M, Grajewski J, Kosicki R, Böhm J, Zentek J (2011). The impact of the Fusarium mycotoxin deoxynivalenol on the health and performance of broiler chickens. Int J Mol Sci.

[42] Dänicke S, Matthes S, Halle I, Ueberschär KH, Döll S, Valenta H (2003). Effects of graded levels of Fusarium toxin-contaminated wheat and of a detoxifying agent in broiler diets on performance, nutrient digestibility and blood chemical parameters. Br Poult Sci.

[43] Grenier B, Bracarense AP, Schwartz HE, Lucioli J, Cossalter AM, Moll WD, Schatzmayr G, Oswald IP (2013). Biotransformation approaches to alleviate the effects induced by fusarium mycotoxins in swine. J Agric Food Chem.

[44] Li XZ, Zhu C, de Lange CF, Zhou T, He J, Yu H, Gong J, Young JC (2011). Efficacy of detoxification of deoxynivalenol-contaminated corn by *Bacillus* sp. LS100 in reducing the adverse effects of the mycotoxin on swine growth performance. Food Addit Contam Part A Chem Anal Control Expo Risk Assess..

[45] Han Z, Willer T, Li L, Pielsticker C, Rychlik I, Velge P, Velgec P, Kaspersd B, Rautenschlein S (2017). Influence of the gut microbiota composition on *Campylobacter jejuni* colonization in chickens. Infect Immun.

